# ﻿A new endemic freshwater mussel (Bivalvia, Unionidae) genus and species, *Ligodontaobscura*, from the Yadkin-Pee Dee River drainage in North Carolina, USA

**DOI:** 10.3897/zookeys.1248.152952

**Published:** 2025-08-07

**Authors:** Michael A. Perkins, Katharine L. DeVilbiss, Nathan V. Whelan, Rachael A. Hoch, Brena K. Jones, Jason W. Mays, Heather K. Evans, Sierra B. Benfield

**Affiliations:** 1 North Carolina Wildlife Resources Commission, Marion, NC 28752, Marion, USA North Carolina Wildlife Resources Commission Marion United States of America; 2 Raleigh, NC, USA Unaffiliated Raleigh United States of America; 3 Southeast Conservation Genetics Laboratory, Warm Springs Fish Technology Center, United States Fish and Wildlife Service, Auburn, AL 36849, Auburn, USA United States Fish and Wildlife Service Auburn United States of America; 4 School of Fisheries, Aquaculture, and Aquatic Sciences, Auburn University, Auburn, AL 36849, Auburn, USA Auburn University Auburn United States of America; 5 North Carolina Wildlife Resources Commission, Raleigh, NC 27601, Raleigh, USA North Carolina Wildlife Resources Commission Raleigh United States of America; 6 North Carolina Wildlife Resources Commission, Creedmoor, NC 27522, Creedmoor, USA North Carolina Wildlife Resources Commission Creedmoor United States of America; 7 United States Fish and Wildlife Service, Asheville, NC 28801, Asheville, USA United States Fish and Wildlife Service Asheville United States of America

**Keywords:** Biodiversity, conservation, phylogenetics

## Abstract

We describe a new genus and species of freshwater mussel, *Ligodontaobscura***gen. et sp. nov.**, using an integrative taxonomic approach consisting of morphological, genetic, biogeographic, and life history information. Specimens of *L.obscura* are poorly represented in historic collections and were infrequently collected; additionally, the species was originally overlooked as a unique taxon due in part to its small size and superficial morphologic similarities to *Lasmigonasubviridis* (Conrad, 1835) and *Alasmidonta* spp. Phylogenetic results place the new species sister to, but consistently distinct from, *Alasmidonta* s.s. We offer a suite of field-ready diagnostics to differentiate *L.obscura* from similar and co-occurring species and briefly discuss the species’ unique phylogenetic placement, which warrants the recognition of a new genus. Importantly, *L.obscura* appears to be a “microendemic” species with a remarkably limited documented range of approximately 13 km within two small, adjacent watersheds of the Yadkin-Pee Dee River drainage. Despite intensive surveys, the extant population is currently known from an approximately 7 km contiguous reach of one stream, making this newly-discovered species a high conservation priority.

## ﻿Introduction

Freshwater mussels (Bivalvia, Unionidae) are among the world’s most threatened faunal groups (Haag and Williams 2014; Lopes-Lima et al. 2018). As the conservation needs for this group continue to grow, integrative taxo­nomic approaches and phylogenetics have emerged as useful tools for effective management (Perkins et al. 2017; Lopes-Lima et al. 2021; Lyubas et al. 2022; Sano et al. 2022). This relatively recent focus on evidence-based taxonomy has resulted in an explosion of research, often leading to the formal description of overlooked, under-researched, and/or cryptic mussel taxa (Keogh and Simon 2019; Liu et al. 2023; Whelan et al. 2023).

The southeastern United States supports the second-most biodiverse freshwater mussel assemblage globally, including nearly 100 imperiled species within the major diversity “hotspots” of the Mississippi, Tennessee, and Mobile River drainages (Haag 2010; Graf and Cummings 2021). In contrast, Atlantic Slope drainages are relatively species-poor with fewer instances of narrow endemism. However, recent research has suggested that some areas may harbor higher instances of endemism than previously thought, such as the geologically unique Uwharrie region of North Carolina (Whelan et al. 2023), the location of the present study.

Freshwater mussel-shell morphology is notoriously variable even among individuals of the same species, with many variables thought to influence morphological phenotypes such as sex, developmental characteristics, and landscape-scale abiotic factors (e.g. water chemistry, stream order, substrate composition; see Haag 2012). This morphological variation has proven to be a significant taxonomic barrier over the last century (Ortmann 1920; Hornbach et al. 2010). Additionally, accurate identification of mussel species is often difficult, sometimes leading to a cascade of problems when tasked with conserving rare or at-risk species (Ferreira-Rodríguez et al. 2019; Bolotov et al. 2024). The freshwater mussel fauna of the Uwharrie region is no exception, with a combination of unique morphologies representing both well-known wide-ranging species such as *Elliptiocomplanata* Lightfoot, 1786 and narrowly endemic species such as the recently described *Alasmidontauwharriensis* (Whelan et al. 2023)

*Lasmigonasubviridis* Conrad, 1835, a species proposed as “Threatened” under the United States Endangered Species Act, is an uncommon but relatively wide-ranging species native to the eastern U.S. from the states of New York south to North Carolina. In North Carolina, the species is distributed in several major river drainages, which includes a seemingly disjunct population in the Uwharrie region of the central Piedmont where putative individuals were sporadically documented from 2004–2010. However, specimens collected in 2016 by the authors lacked the diagnostic morphologic characters common to *L.subviridis* (see “Comparisons” below), and one live individual was retained for species identification via genetic confirmation. The initial genetic results were inconclusive, as this specimen did not appear to closely match any documented taxon. Resequencing of the individual, along with several additional specimens collected during subsequent surveys from 2016–2021 and a preserved individual from the North Carolina Museum of Natural Sciences (NCMNS) mollusk collection, confirmed that the species was not only unknown to science, but apparently restricted to only two small (<4^th^ order) streams in North Carolina.

In this paper, we present a new genus and species of freshwater mussel, *Ligodontaobscura*. We document compelling independent lines of evidence including morphological, genetic, life history, and biogeographic information to support the recognition of this new taxon. We discuss the unique phylogenetic position of *L.obscura* and systematics of the closely related genera *Alasmidonta* Say, 1818 and *Lasmigona* Rafinesque, 1831. Additionally, we provide a useful suite of field-ready diagnostic morphological characters for *L.obscura* that will aid in identification and conservation of this rare species.

## ﻿Materials and methods

### ﻿Surveys, specimen collection, and tissue acquisition.

Freshwater mussel surveys are regularly conducted throughout North Carolina by North Carolina Wildlife Resources Commission (NCWRC) staff and others, such as university groups and environmental consulting firms. The authors have chosen to limit our reporting to timed surveys conducted by trained NCWRC staff and colleagues in the Uwharrie River and Little River watersheds over the period of 2016–2022. Two individuals of the undescribed species were preserved in the field with 95% non-denatured ethyl alcohol, relic shells were stored in zipped plastic bags, and eight live individuals were transported to the NCWRC Marion Conservation Aquaculture Center (MCAC) in North Carolina for life history studies. Tissue and shell materials were also acquired under loan from the NCMNS mollusk collection. For most individuals, 9 mm Isohelix DNA Buccal Swabs were used to collect DNA from the foot tissue of a subset of specimens. After sample collection, swabs were stored and preserved in 5 ml plastic cryo vials containing either 95% non-denatured ethyl alcohol or Buccalfix® DNA Stabilization Buffer (IsoHelix; Cell Projects Ltd). Morphological inspections and dissections were performed by eye or with the aid of a stereoscopic microscope, and measurements were taken with digital calipers.

### ﻿DNA extraction, amplification, and sequencing.

DNA samples were processed at either the US Fish and Wildlife Service Southeast Conservation Genetics Lab (SECGL) in Auburn, Alabama or the NCMNS. DNA was extracted from swabs using the IsoHelix Xtreme DNA Isolation kit (Cell Projects Ltd) following manufacturer’s instructions. For swabs placed in ethanol, swabs were first removed and then placed in lysis buffer with proteinase K. For swabs placed in stabilization buffer, proteinase K was added directly to the buffer to start DNA extractions. For tissue-based extractions, the DNeasy Blood and Tissue Kit (QIAGEN) was used following manufacturer’s instructions.

Three genes were sequenced for phylogenetic analyses: 1) mitochondrial cytochrome *c* oxidase subunit 1 (COI), 2) mitochondrial NADH dehydrogenase subunit 1 (ND1), 3) nuclear internal transcribed spacer I (ITS1). PCR conditions for each gene processed at SECGL followed Whelan et al. (2023). PCR products were cleaned using the New England Biolabs Monarch PCR Cleanup kit and sent to Genewiz for Sanger sequencing in both directions using the same primers as those used for PCR amplification. PCR protocols for samples processed at NCMNS were similar. COI was amplified using 1 µL M13 tailed Folmer primers (Folmer et al. 1994), 5 µl of a 1:10 mixture of Takara Ex Taq and Promega GoTaq, 0.6 µL 25 mM MgCl_2_ and one µL DNA in a 10 µL reaction. Cycling conditions were 94 °C for 2 min followed by 35 cycles of 94 °C for 30 s, 46 °C for 30 s, and 72 °C for 45 s with a final 10 min elongation step at 72 °C. ND1 was amplified similarly using either M13 tailed LeuF 5’-TTG GCAGAAAGTGCATCAGATTA-3’ and LoGlyR 5-CCTGCTTGGAAGGCAAGTGTACT-3’ or M13 tailed ND1-F 5’-GCTATTAGTAGGTCGTATCG-3’ (Buhay et al. 2002) and ND1-R 5’-GCTATTAGTAGGTCGTATCG-3’ (Serb et al. 2003) with a 50 °C annealing step and a 1 min extension. ITS was similarly amplified using primers published in King et al. (1999), 54 °C annealing and 1 min extension. All primers were concentrated at 10 µM. Products were cleaned using ExoSAP-IT (Thermofisher). COI and ND1 products were sequenced with M13 primers and ITS was sequenced using the same PCR primers. Sequencing was carried out in 10 µL reactions using 1.2 µL DNA, 0.3 µL primer, 0.25 µL BigDye Enhancing Buffer (McLab), 0.125 µL BigDye (Thermofisher), and 1.75 µL 5X Sequencing Buffer (McLab). Sequencing products were cleaned via ethanol precipitation and run on an ABI 3500. All raw chromatograms were visualized in Geneious (Dotmatics) and manually checked for sequencing errors.

Other *Lasmigona* and outgroup sequences of the three genes sequenced here were downloaded from GenBank (Suppl. material 1: table S1). Outgroups were chosen based on Lopes-Lima et al. (2018) and mostly followed Whelan et al. (2023). For phylogenetic analyses with multiple genes, sequences with enough metadata to determine they were from the same individual were combined. For some outgroup samples, sequences from different individuals of the same species were combined to create chimeric individuals, which was done to maximize character sampling while minimizing missing data (see Whelan et al. 2023; Suppl. material 1: table S1).

### ﻿Phylogenetics

Protein coding mitochondrial genes were aligned with Clustal Omega 1.2.2 using default parameters (Sievers et al. 2011). ITS1 was aligned with MAFFT using the L-INSI algorithm and default parameters (Katoh and Standley 2013). Alignments were concatenated with Geneious to create a mitochondrial dataset, a reduced mitochondrial dataset that had non-*Lasmigona* individuals without both mitochondrial genes removed, and a combined ITS plus reduced mtDNA dataset that excluded any non-*Lasmigona* without all three genes except *A.viridis* and *A.heterodon*, which were retained to maximize taxon sampling despite having only one or two genes sequenced (Suppl. material 1: table S1). Some *L.obscura* samples were not concatenated for analyses, but this did not influence overall results (Table 1).

**Table 1.** GenBank accession numbers for sequences generated here. Information for all taxa can be found in Suppl. material 1: table S1.

Species	GenBank description and taxon name in trees and alignments	ND1	COI	ITS
*Ligodontaobscura*	Ligodonta_obscura_USNM1607186	PV734019	PV738973	
*Ligodontaobscura*	Ligodonta_obscura_NCSM22120	PV734020	PV738974	
*Ligodontaobscura*	Ligodonta_obscura_249^†^	PV734024	PV738968*	PV739327
*Ligodontaobscura*	Ligodonta_obscura_251^†^	PV734017	PV738970*	
*Ligodontaobscura*	Ligodonta_obscura_252^†^	PV734025	PV738969*	PV739322
*Ligodontaobscura*	Ligodonta_obscura_253^†^	PV734026	PV738962	PV739321
*Ligodontaobscura*	Ligodonta_obscura_268^†^	PV734018	PV738963	PV739323
*Ligodontaobscura*	Ligodonta_obscura_270^†^	PV734027	PV738964	
*Ligodontaobscura*	Ligodonta_obscura_271^†^	PV734028		
*Ligodontaobscura*	Ligodonta_obscura_272^†^	PV734023	PV738965	PV739325
*Ligodontaobscura*	Ligodonta_obscura_NCSM221197.2	PV734012		PV739318
*Ligodontaobscura*	Ligodonta_obscura_NCSM221197.1	PV734016		PV739319
*Ligodontaobscura*	Ligodonta_obscura_NCSM221198	PV734013		
*Ligodontaobscura*	Ligodonta_obscura_NCSM221197.3	PV734014		PV739320
*Ligodontaobscura*	Ligodonta_obscura_D120	PV734015		
*Ligodontaobscura*	Ligodonta_obscura_Lasu1^†^	PV734022	PV738966*	PV739326
*Ligodontaobscura*	Ligodonta_obscura_Lasu2^†^	PV734021		PV739324
*Ligodontaobscura*	Ligodonta_obscura_Lasu3^†^		PV738967*	PV739328
*Ligodontaobscura*	Ligodonta_obscura_NCSM_47145		PV738972	
*Lasmigonadecorata*	Lasmigona_decorata_CAC15	PV734010	PV738975	PV739329
*Lasmigonadecorata*	Lasmigona_decorata_CAC16		PV738976	PV739330
*Lasmigonadecorata*	Lasmigona_decorata_CAC17	PV734011	PV738977	PV739331

*These sequences were kept separate (i.e. not concatenated) in phylogenetic analyses. †These sequences were collected from specimens in the field.

Best-fit substitution models and partitions for phylogenetic inference were inferred with ModelFinder (Kalyaanamoorthy et al. 2017) as implemented in IQ-TREE 1.6.12 (Nguyen et al. 2015). For partition finding with mitochondrial genes, codon positions were used as starting blocks. Partition finding with ITS used the whole gene as a starting block. All model testing used linked branches, a unique evolutionary rate for each partition, and Bayesian information criteria for determining the best-fit model and partitioning scheme. After model-testing, best-fit models and partitions were used in maximum-likelihood tree inference with IQ-TREE. Tree searches used default parameters except perturbation strength was set to 0.2 and 500 iterations had to be unsuccessful to stop tree inference. Tree inference was done 20 times for each dataset. Only the tree with the best log-likelihood score was retained. Support for relationships was measured with 1,000 ultrafast bootstrap replicates (Hoang et al. 2018).

Outgroups were chosen based on Lopes-Lima et al. (2018) and mostly followed Whelan et al. (2023). Outgroup sequences of the three genes sequenced here were downloaded from GenBank (Suppl. material 1: table S1). For phylogenetic analyses with multiple genes, sequences with enough metadata to determine they were from the same individual were combined. In some cases, for outgroup samples, sequences from different individuals of the same species were combined to create chimeric individuals, which was done to maximize character sampling while minimizing missing data.

### ﻿Abbreviations

**NCMNS** North Carolina Museum of Natural Science

**NCSM** identifier code in Global Registry for Scientific Collections for material collections stored at the NCMNS

**NCWRC** North Carolina Wildlife Resources Commission

**MCAC** Marion Conservation Aquaculture Center

**USFWS** United States Fish and Wildlife Service

## ﻿Results

### ﻿Surveys

**Figure 1.**
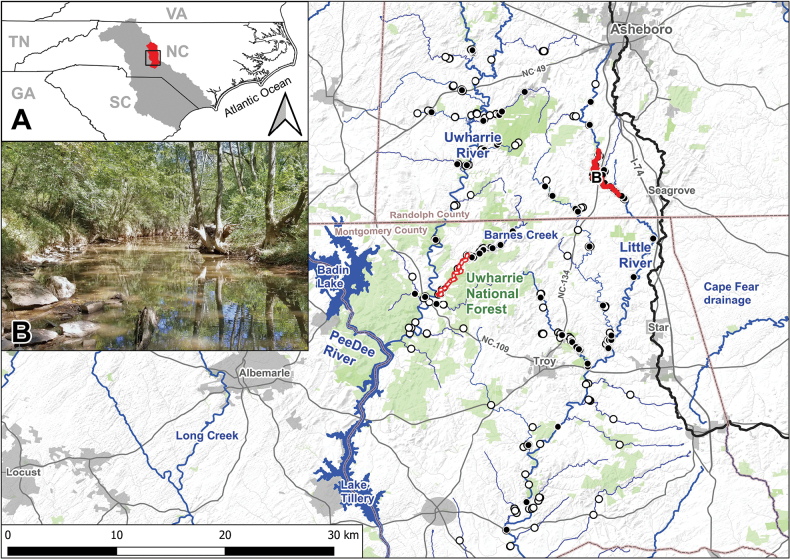
Terrain map of survey locations and the known range of *Ligodontaobscura*. Red stream segments indicate the range of the species from continuous element occurrence data (NCWRC and NCSM records) with ~1 km up and downstream buffers; the continuous red line represents the current range within Little River, the dashed red line represents the potential range within Barnes Creek. Black circles indicate recent survey locations (2016–2022) in the Uwharrie and Little River watersheds where *L.obscura* was not encountered, open circles indicate additional historic survey records (before 2016) where the species was not encountered. Green shading represents conserved or protected land boundaries, gray shading represents municipal boundaries. A. The boundaries of North Carolina and surrounding states; gray shading represents the Yadkin-PeeDee River drainage and red shading represents the Uwharrie River and Little River watersheds; B. An example of typical habitat preferred by the species in Little River near the type locality (24 September 2019, photo credit: M.A. Perkins).

A total of 141 surveys were conducted (Fig. 1) with approximately 610 person-hours of search effort. The undescribed species was encountered at 11 unique sites; a total of 43 live individuals and five shells were collected. Catch-per-unit-effort (CPUE; an indirect measure of species abundance calculated by dividing the number of target individuals encountered by the search effort) among all sites was low (CPUE = 0.07 individuals per person-hour). Put another way, an estimated 14.2 hours of survey effort were required to locate one individual. To date, the undescribed species has only been detected in contemporary surveys within the mainstem of the Little River in Randolph County, North Carolina. However, given well-known, low detection rates of freshwater mussels in general (Huang et al. 2011; Reid 2016), it is possible that the species could exist in additional waters. For example, we hypothesize that the species could occur in additional downstream reaches, the adjacent Barnes Creek (surveyed for 105 person hours compared to 280 person hours in the mainstem Little River, and with 3 individuals of the species previously collected prior to this study; see “Material examined” and “Distribution”) or in some tributaries to the Little River.

### ﻿Genetic data generation and phylogenetics

Genetic data were generated for 24 individuals of the previously undescribed species Table 1). We also sequenced three specimens of *Lasmigonadecorata* Lea, 1852 from the Yadkin-Pee Dee basin (Table 1). Numerous individuals had shorter COI and ND1 sequences than the entire alignment length, mostly as a result of only shorter sequence fragments being available from GenBank, but also because of some difficulties sequencing individuals sampled here. As such, missing data for COI and ND1 were exclusively at the 5’ and 3’ end of the alignments. Sequencing for ITS1 sometimes partially failed, resulting in small fragments for some individuals. Sequencing failures for ITS1 are attributable to one or more microsatellite regions that made Sanger sequencing difficult (see also Whelan et al. 2023). The number of individuals in each dataset ranged from 232 in the mtDNA_ITS dataset to 535 in the COI_ND1 dataset (Table 2). The COI dataset had the least amount of missing data, whereas the primary dataset used for conclusions, “mtDNA_ITS” had a moderate amount of missing data (23.7%), mostly as a result of ITS alignment gaps and ITS sequences and mitochondrial sequences from GenBank being shorter than the targeted gene fragments (Table 2).

**Table 2.** Molecular dataset characteristics.

Dataset	Number of individuals	Alignment Length	Missing Data %
COI	449	658	6.4
ND1	409	876	14.0
COI_ND1	535	1534	28.9
mtDNA_reduced	350	1534	13.2
ITS	244	682	29.8 (including gaps)
mtDNA_ITS	232	2216	23.7 (including gaps)

**Figure 2.**
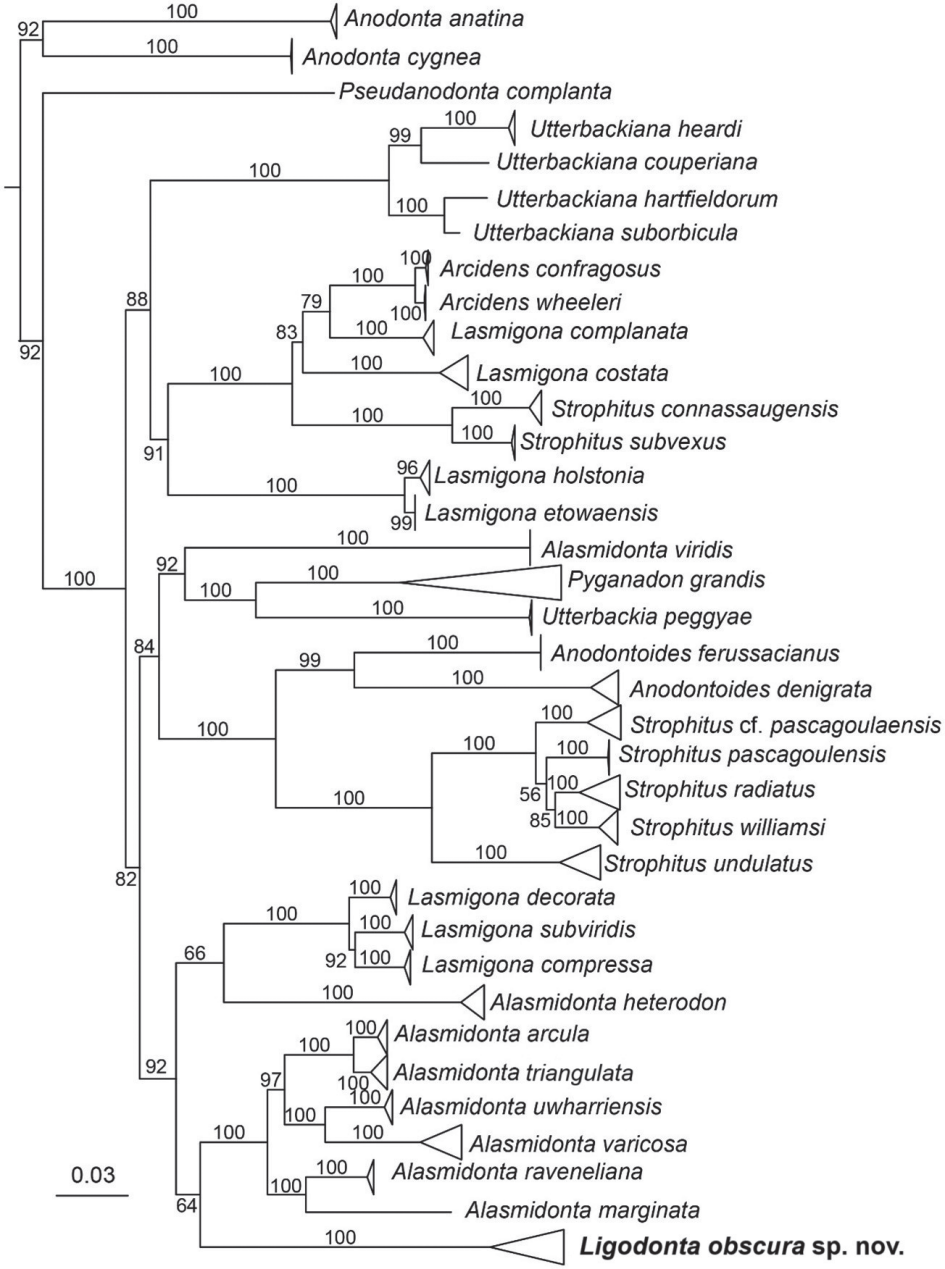
Concatenated mtDNA and ITS1 phylogenetic tree from maximum-likelihood analysis in IQ-Tree. Terminal tips collapsed to aid visualization; the full tree is available as Suppl. material 1. Nodes are labeled with ultrafast bootstrap support. Scale bar represents substitutions per site. *Ligodontaobscura* sp. nov. is labeled in bold.

Phylogenetic results generally mirrored those from past studies for examined taxa (Smith et al. 2018; Whelan et al. 2023), but the undescribed species was on a distinct branch in every analysis. All individuals of the undescribed species were united with 100 UFboot support in every analysis (Fig. 2; Suppl. material 1). The sister clade to the undescribed species was uncertain despite robust taxon sampling, but most analyses placed the lineage sister to *Alasmidonta*, *sensu stricto* (Fig. 2, Suppl. material 1). That is the undescribed species was sister to *Alasmidonta* s.s. on the COI, COI_ND1, mtDNA_reduced, and mtDNA_ITS trees with varying levels of support (UFboot = 64–92%). On the ND1 tree, the undescribed species was sister to the clade containing *L.decorata*, *L.subviridis*, and *L.compressa* with moderate support (89% UFboot; Suppl. material 1). On the ITS tree, the undescribed species was sister to a clade containing *L.decorata* and *L.subviridis* with moderate support (84% UFboot; Suppl. material 1).

The undescribed species is not closely related to *Lasmigonas.l.* and is a distinct lineage from *Alasmidontas.s.* The genera *Alasmidonta* and *Lasmigona*, as currently recognized, are not monophyletic and our results corroborate previous analyses of these groups (Whelan et al. 2023). Broadly, our results do not provide conclusive support for the sister group of the undescribed species. However, our data firmly rejects placing the undescribed lineage into a currently recognized genus. Thus, we have proposed the nomen *Ligodonta* gen. nov. in recognition of this unique lineage.

### ﻿Systematics

**Class Bivalvia Linnaeus, 1758**

**Order Unionida Stoliczka, 1871**

**Family Unionidae Rafinesque, 1820**

**Tribe Anodontini Rafinesque, 1820**

#### ﻿Genus Ligodonta

Taxon classificationAnimaliaUnionidaUnionidae

Perkins, DeVilbiss & Whelan, 2025 gen. nov.

268073B0-9146-544E-972E-565C764967A0

https://zoobank.org/BA90D85A-A158-48FD-8CB5-27DC57046EEC

##### Type species.

*Ligodontaobscura* Perkins, DeVilbiss & Whelan, 2025, sp. nov.

##### Diagnosis.

Monotypic, see species Diagnosis below.

##### Etymology.

Greek, *ligo*- meaning “little” and -*donta* meaning “tooth.”

##### Species included.

*Ligodontaobscura* Perkins, DeVilbiss & Whelan, 2025, sp. nov.

#### ﻿ Ligodonta obscura

Taxon classificationAnimaliaUnionidaUnionidae

Perkins, DeVilbiss & Whelan, 2025 sp. nov.

F5F475F6-74C1-527C-A9EC-D4B75BAFC3AD

https://zoobank.org/C18E48EB-120A-4290-876C-F496D66EFE27

##### Etymology.

*obscura* from English “obscure” in reference to the species evading discovery until the present study.

##### Proposed common name.

Solstice creekmussel, in reference to the species’ reproductive period centered around the winter solstice and preferred habitat of small creeks.

##### Type.

**Figure 3.**
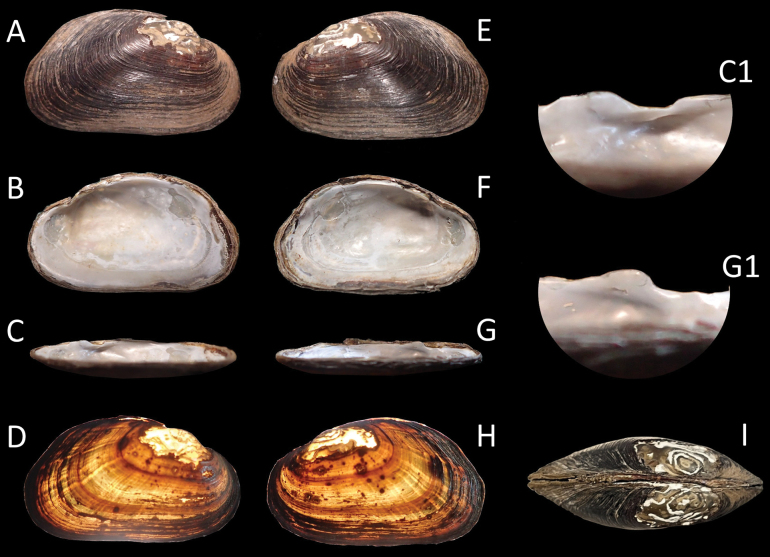
*Ligodontaobscura*, Holotype NCSM 221198. A. Right valve exterior view; B. Right valve interior view; C. View of right valve dentition profile from ventral margin **C1** detail of right valve pseudocardinal tooth; D. Right valve exterior view with backlight; E. Left valve exterior view; F. Left valve interior view; G. View of left valve dentition profile from ventral margin **G1** detail of left valve pseudocardinal tooth; H. Left valve exterior view with backlight; I. Ligament view with posterior facing left. Specimen length = 35.8 mm.

***Holotype*.** • NCSM 221198 (Fig. 3A–I): Little River adjacent to NC 134; coordinates withheld by authors; collectors: BK Jones, KL DeVilbiss, MA Perkins, MA Burchfiel, JK McIver; 24 September 2019. Shell and soft tissues in 95% non-denatured ethanol, body removed by cut of adductor muscles and separation of mantle from the shell. Description as above in Diagnosis. Shell length = 35.8 mm, shell width = 13.2 mm, shell height = 21.0 mm. GenBank PV734013.

**Figure 4.**
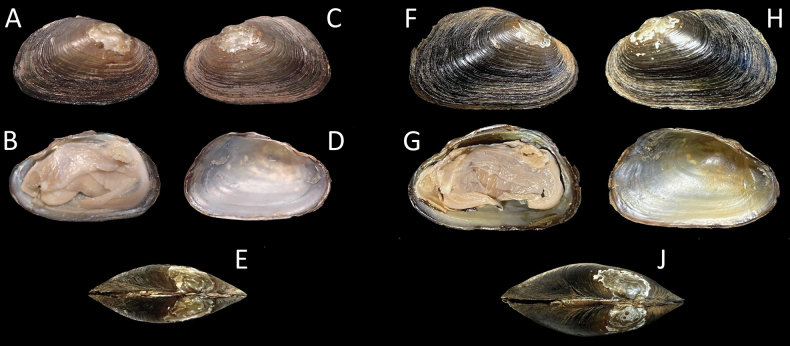
*Ligodontaobscura*, paratypes. A–D. NCSM 47145; A. Right valve exterior view; B. Right valve interior and soft tissues; C. Left valve exterior view; D. Left valve interior; E. Ligament view with posterior facing left. Specimen length = 27.3 mm; E–H. USNM 1607186; F. Right valve exterior view; G. Right valve interior and soft tissues; H. Left valve exterior view; I. Left valve interior; J. Ligament view with posterior facing left. Specimen length = 34.2 mm.

***Paratypes*.** • USNM 1607186 (Fig. 4A–E): Little River adjacent to NC 134; coordinates withheld by authors; collectors: KL DeVilbiss, JK McIver, SM Sheats, JW Mays, K Saxton; 18 June 2019. Shell and soft tissues in 95% non-denatured ethanol, body removed by cut of adductor muscles and separation of mantle from the shell. Description as above in Diagnosis. Shell length = 34.2 mm, shell width = 12.4 mm, shell height = 20.1 mm. GenBank PV34019, PV738973. • NCSM 47145 (Fig. 4F–J): Barnes Creek in the vicinity of SR 1303 crossing; coordinates withheld by authors; 1 whole animal in 95% ethanol; collectors: CB Eads, RA Harrington; 10 March 2010. Soft tissues detached from left valve by cut of adductor muscles and separation of mantle from the shell. Description as above in Diagnosis. Shell length = 27.3 mm, shell width = 9.6 mm, shell height = 16.2 mm. GenBank PV738972.

##### Other material examined

**Figure 5.**
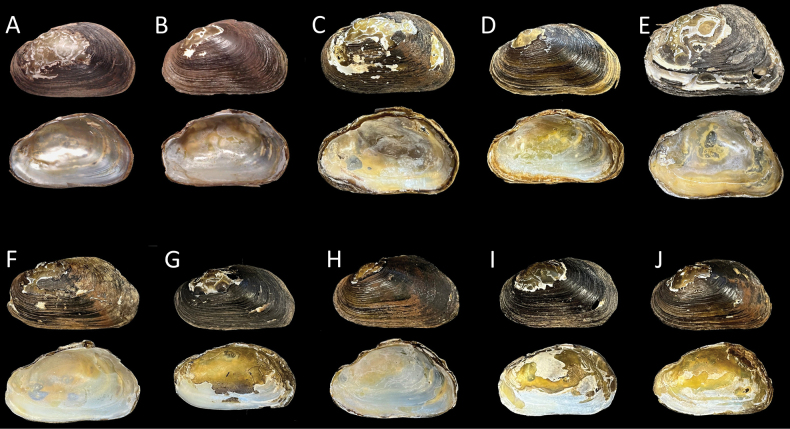
*Ligodontaobscura*, other material examined. Specimens in upper row, columns A–E with soft tissues (*) and/or genetic data (**^†^**) available (Table 1); specimens in lower row, columns F–J consist of dry valves only. Photos of left valve exterior and right valve interior. A. NCSM 29612*, length *(l* = 37 mm; B. NCSM 221197.1**^†^**, *l* = 33.4 mm; C. NCSM 221197.2**^†^**, *l* = 39.4 mm; D. NCSM 221197.3**^†^**, *l* = 37.2 mm; E. NCSM 221201*^†^, *l* = 39.2 mm; F. NCSM221200, *l* = 46.1 mm; G. NCSM 221199, *l* = 46.2 mm; H. USNM 1607185, *l* = 35.5 mm; I. 210618.1kld, *l* = 33.6 mm; J. NCSM221195, *l* = 34.7 mm.

**(Fig. 5A–J).** North Carolina: Randolph County. • 1) NCSM 221197—Little River adjacent to NC 134; coordinates withheld by authors; 3 paired dry bivalves with tags D116, D117, D118; collectors: BK Jones, KL DeVilbiss, MA Perkins, MA Burchfiel, JK McIver; 24 September 2019. • 2) NCSM 221196—Little River in the vicinity of SR 1127 crossing; coordinates withheld by authors; 1 paired dry bivalve; collectors: KL DeVilbiss, CL Lynch; 18 June 2021. • 3) NCSM 221195—Little River adjacent to NC 134; coordinates withheld by authors; 1 paired dry bivalve; collectors: KL DeVilbiss, CL Lynch, RA Hoch; 21 September 2021. • 4) NCSM 221199—Little River in the vicinity of SR 1121 crossing, coordinates withheld by authors; 2 paired dry bivalves; collectors: RJ Heise, MA Perkins, WT Russ, W Xiong, SL Stevens; 2 June 2016. • 5) NCSM 221200—Little River in the vicinity of SR 1135 crossing; coordinates withheld by authors; 1 paired dry bivalve; collectors: BK Jones, RJ Heise, TR Fox, RA Hoch, JL Griffin; 20 April 2010. • 6) NCSM 221201—Little River, coordinates withheld by authors; 1 whole animal in 95% ethanol; collectors: KL DeVilbiss, JK McIver, SM Sheats, JW Mays, K Saxton; 18 June 2019. Montgomery County. • 7) NCSM 47155—Barnes Creek in the vicinity of SR 1134 crossing; coordinates withheld by authors; 1 paired dry bivalve; collectors: CB Eads, RA Harrington; 1 April 2010. • 8) NCSM 29612—Barnes Creek in the vicinity of SR 1303; coordinates withheld by authors; 1 whole animal in 95% ethanol; collectors: CB Eads, P Hubert, E Schubert; 29 March 2004.

##### Diagnosis.

**Figure 6.**
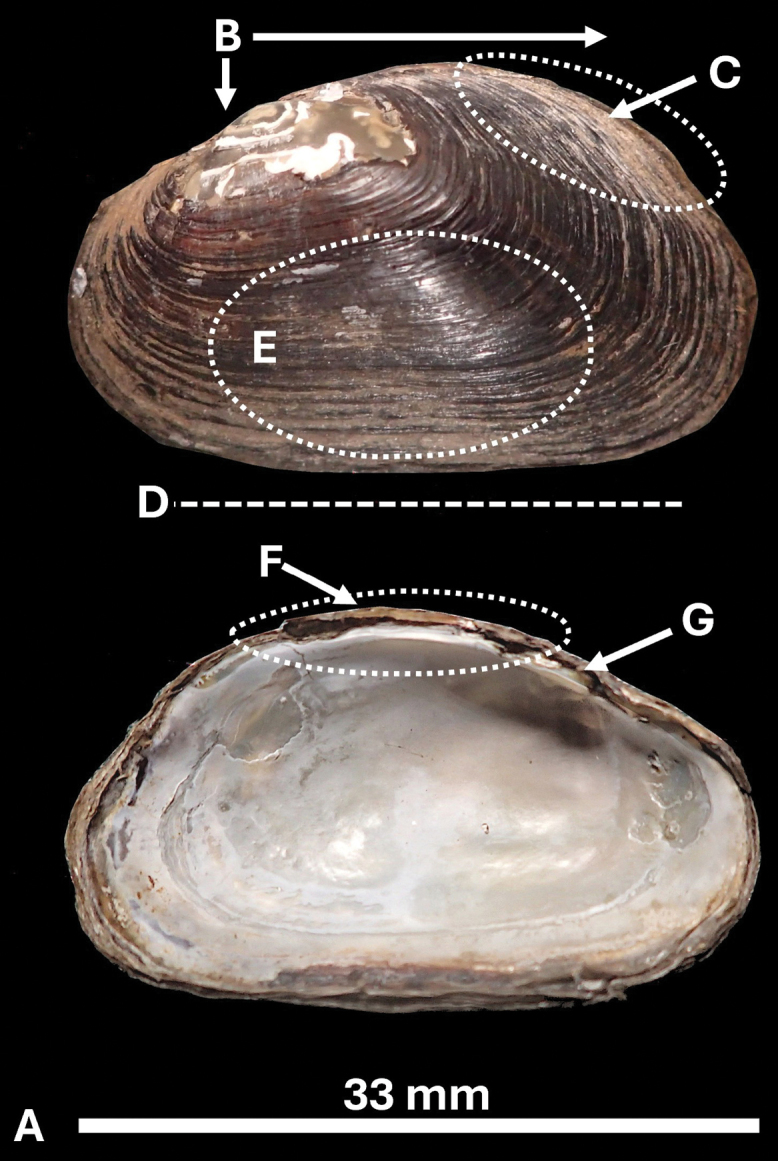
Morphological diagnosis of *Ligodontaobscura* shell characters. A. Length does not exceed 50 mm; B. Umbo slightly above hingeline, posterior ridge higher than umbo; C. Posterior slope lacking corrugations; D. Ventral margin nearly straight; E. Periostracum dark, thin, papery; F. Lateral teeth absent; G. Pseudocardinal teeth greatly reduced or rudimentary. Holotype specimen NCSM 221198.

Shell morphology (Figs 3–6, 10). Shell small, thin, subovate, and moderately inflated; umbo extending slightly beyond the hinge line, with single or weakly double-looped beak sculpture in younger individuals; hinge weak. Anterior laterally compressed; anterior margin strongly rounded towards ventral margin. Ventral margin straight. Posterior margin broadly rounded, higher than umbo distally. Posterior ridge weakly pronounced, rounded, double. Periostracum somewhat thin, papery, appearing dark brown in most individuals; surface with fine, minor growth lines. The following color description was aided by backlight (Fig. 3D, H): periostracum interrupted by dark concentric growth rings, base color yellow or light brown, anterior with variably spaced dark green or brown rays.

Nacre diaphanous, somewhat iridescent with blue or cream tint. Umbo cavity shallow. Anterior adductor scar well defined; posterior adductor scar weak. In the left valve, a single, small, smooth, laterally-compressed pseudocardinal tooth extending to a weak interdental projection, oriented directly above the umbo cavity and parallel to the hinge line. Left lateral tooth absent, appearing as a weakly inflated ridge. In the right valve, a single small, rounded, laterally compressed, triangular pseudocardinal tooth oriented anterior to the umbo cavity and parallel to the anterior margin. Right lateral tooth as described above. No obvious sexual dimorphism in shell morphology.

General external characteristic measurements as follows (*n* = 14): shell length = 23.2 mm min – 46.2 mm max, *x̄* = 35.9 mm; shell height = 14.6 mm min – 26.3 mm max, *x̄* = 20.7 mm; shell width 7.3 mm min – 17.5 mm max, *x̄* = 13.8 mm; ratios from averages as follows: length 1.73 times height and 2.59 times width, height 1.5 times width.

**Figure 7.**
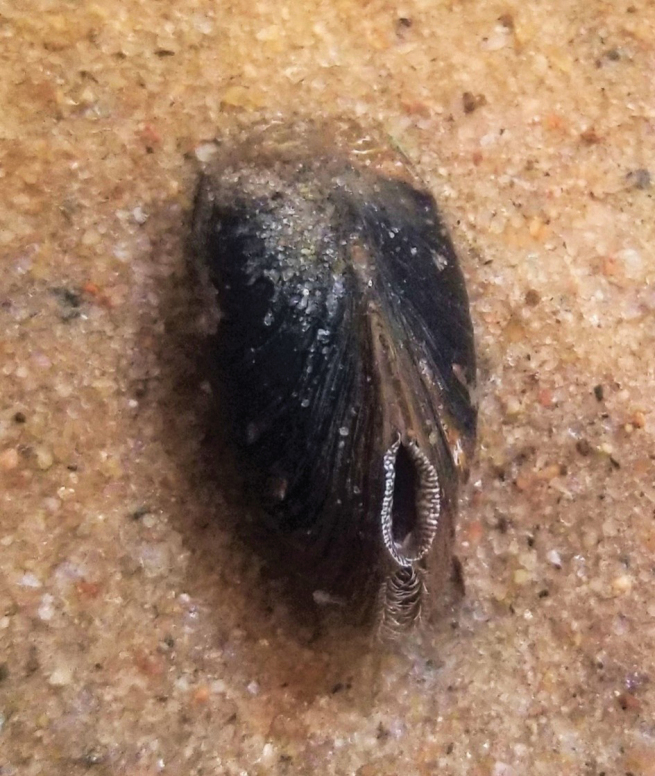
*Ligodontaobscura* in life. Live specimen held at the Marion Center for Aquatic Conservation in January 2022. Holotype specimen NCSM 221198.

Soft anatomy (Figs 4B, F, 7). Incurrent and excurrent apertures approximately equal in length, separated by thin mantle bridge. Excurrent aperture lacking papillae, smooth or weakly crenulate, dark gray to dark brown, occasionally with irregular cream-colored stripes. Incurrent aperture papillae present, simple or sometimes bifurcate anteriorly, dark gray to dark brown at base and cream-colored to tan distally (Fig. 7). Posterior margin of mantle pigmented, appears as a thin, dark band anterior to apertures. Inner lamellae of gills fully attached to visceral mass; outer gills marsupial. Foot tissue pale pink-orange.

**Figure 8.**
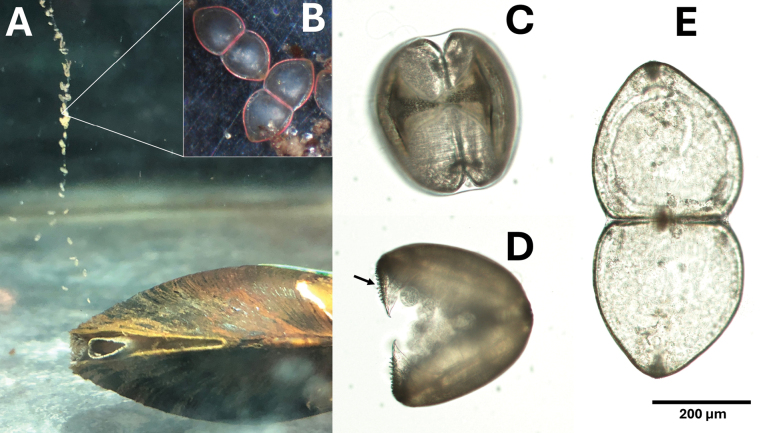
Glochidia release and glochidia morphology. A. Glochidia release by a gravid female *Ligodontaobscura* under laboratory conditions, white arrow denotes larval glochidia attached to a thin mucus thread being released from the excurrent aperture; B. Glochidia occasionally have a pink or red hue; C. Ventral view of mature glochidia; D. Lateral view of mature glochidia, black arrow indicates styliform hook with microstylets; E. Open glochidia shell illustrating subtriangular shape.

Glochidia (Fig. 8B–E). Larvae subtriangular (Hoggarth 1999); length approximately 300–350 µm; dorsal margin straight, ventral margin rounded, valves convex; apical styliform hooks present and conspicuous, approximately 50 µm in length and not strongly recurved, mesial surface of hooks with scattered microstylets. Occasionally appearing with a red tint. Charged gills in brooding females pink.

##### Distribution.

*Ligodontaobscura* is a rare mussel with an extraordinarily narrow distribution currently known from only two streams in central North Carolina, USA (Fig. 1). A total of 43 live individuals were observed in 2016–2022 from one contiguous 7.15 km reach in Little River, a direct tributary on the eastern edge of the Pee Dee River in Randolph County. Historical collections are represented by only three specimens at NCSM from an approximately 6 km reach of Barnes Creek, a tributary draining to the Uwharrie River (a major tributary to the Pee Dee River) in Montgomery County, NC. Despite intensive surveys, no live specimens have been recovered from Barnes Creek since 2010, but the species may continue to persist. *Ligodontaobscura* is not currently known from any other locations locally or globally, nor is the species known to be represented in additional museum collections.

##### Life history.

Occupied habitat. *Ligodontaobscura* was found in composite sand/gravel substrates among cobble (mid-channel) and in soft silt (side banks). Individuals were observed lying entirely on top of, minorly buried in, or mostly buried in the river-bottom substrate. River runs and shallow pools were the most common habitat, with a few individuals detected in swift moving riffle habitat (Fig. 1B). Occupied habitat also often included a fine layer of benthic sediment.

##### Reproduction.

*Ligodontaobscura* is presumably tachytictic, becoming gravid around the autumnal equinox and releasing glochidia in winter, shortly after the solstice. Two individuals in long-term holding at MCAC became gravid in late September; one individual slowly (~2 weeks) released approximately 5,500 mature glochidia in late December and early January. *Ligodontaobscura* does not appear to utilize conspicuous mantle adaptations or conglutinate morphology to attract hosts. Glochidia are released through the excurrent aperture directly into the water column and are attached to a thin mucus thread (Fig. 8A).

##### Age.

Inspection of growth rings suggests *L.obscura* is a relatively short-lived species; examined individuals attained a maximum age of 9 or 10 years.

##### Remarks.

Morphological comparisons.

**Figure 9.**
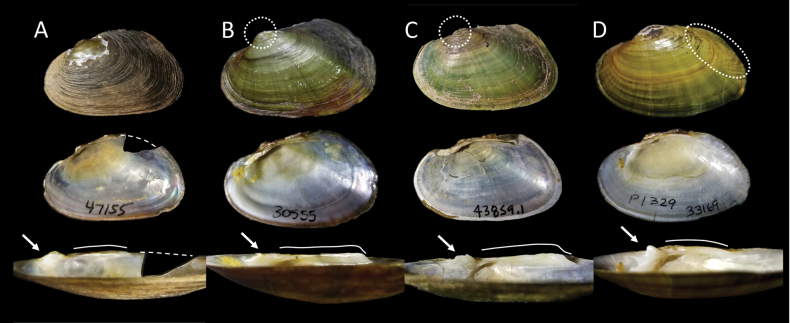
Comparisons of *Ligodonta* with other species. Top row illustrates some external shell characteristics of the left valve for select specimens (all specimens ~25–30 mm in length): column A. *Ligodontaobscura* (NCSM 47155); B. *Lasmigonasubviridis* (NCSM 30555); C. *Lasmigonadecorata* (NCSM 43859.1); D. *Alasmidontauwharriensis* (NCSM 33164). Of note are double-looped beak structure (dashed circles) in specimens B and C, and corrugations along the posterior slope (dashed oval) in specimen D, neither of which are characteristic of *L.obscura* (see “Comparisons” section in text). Middle row illustrates internal shell characteristics of the right valve; *L.obscura* lacks the lateral teeth characteristic of *Lasmigona* specimens B and C, and the pronounced interdental projection of D. Bottom row illustrates dentition characters of the right valve; arrows highlight rounded pseudocardinal teeth in specimens A and D (although D with more prominent projection), sculptured articulated dentition in B and C; drawn lines highlight lateral dentition profile: short and inflated in specimens A and D, long, thin and prominent in specimens B and C.

*Ligodontaobscura* is morphologically similar to several freshwater mussel species in North Carolina waters, specifically those within the genera *Lasmigona* and *Alasmidonta* (Fig. 9). However, this species is distinguishable using a combination of readily identifiable external and internal shell characteristics, life-history traits, and biogeographic information.

**Figure 10.**
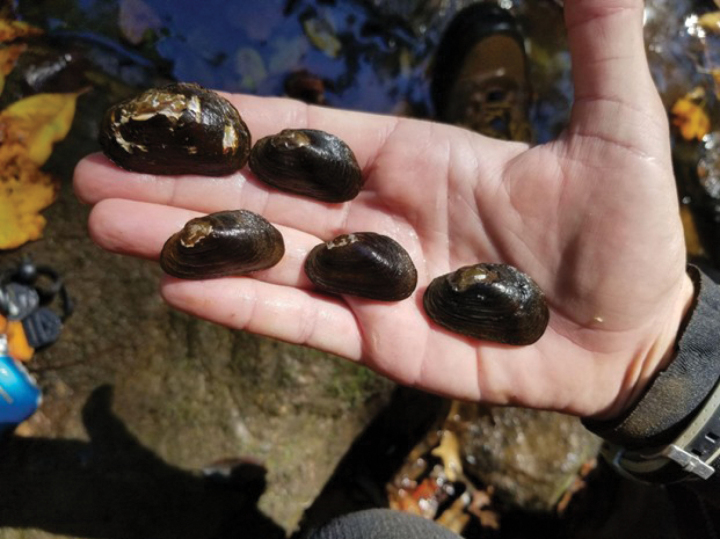
Five live individuals of *Ligodontaobscura* collected from the type locality, 24 September 2019. Photo credit: Michael A. Perkins, NCWRC.

The exterior shell morphology of *L.obscura* is most similar to *Lasmigonasubviridis*, a rare species (recently proposed as federally “Threatened”) with a patchy distribution in predominately mid-Atlantic state waters, including the Roanoke, New, Tar, and Neuse River drainages in North Carolina. Unlike *L.obscura*, *L.subviridis* has a smooth periostracum, rounded ventral margin, typically straight posterior margin angled ~30° above the hingeline, conspicuously double-looped beak sculpture, is generally more laterally inflated, and commonly exceeds the maximum recorded size for *L.obscura*. Internal shell characters are not similar to *L.obscura*; *L.subviridis* possesses strong lateral teeth in both valves, a thickened and weakly serrated pseudocardinal tooth on the right valve, and two conspicuous serrated pseudocardinal teeth on the left valve. These species also differ in life histories; *L.subviridis* typically prefers larger rivers or streams and glochidia commonly undergo direct transformation without an obligate fish host. *Ligodontaobscura* is not known to be sympatric with *L.subviridis*, and *L.subviridis* has not been recovered in the Yadkin-Pee Dee drainage in at least a century.

*Lasmigonadecorata* (Lea, 1852) is an exceptionally rare protected species (federally listed as “endangered”) with a restricted distribution in North and South Carolina with a similar appearance to both *L.obscura* and *L.subviridis*. Exterior and internal shell characteristics of *L.decorata* closely resemble *L.subviridis*. Therefore, *L.decorata* can be differentiated from *L.obscura* by possessing a smooth periostracum, more-pronounced posterior margin, well-developed pseudocardinal and lateral teeth, and attaining a larger size. Distinguishing *L.decorata* from *L.obscura* is simplified by the fact that there is no known overlap in their native ranges. *Lasmigonadecorata* is restricted to only a few Yadkin-Pee Dee and Catawba basin streams in North Carolina, which is west of the known range for *L.obscura*.

While there are exceptions, *Alasmidonta* is often characterized by poorly developed or absent lateral teeth and weakly developed or smooth pseudocardinal teeth. Several species of *Alasmidonta* are known from North Carolina, including the recently described *A.uwharriensis* (Whelan et al. 2023). Even though *A.uwharriensis* is sympatric with *L.obscura*, they are easily differentiated by external shell morphology. *Alasmidontauwharriensis*, and the closely related and comparatively wide-ranging *A.varicosa* (Lamarck, 1819), often possess a bright orange foot, conspicuous corrugations along the posterior slope, the umbo sits above the posterior margin, and the shell is more ventrally inflated and attains a larger size than *L.obscura*. Internal shell characters of *A.varicosa* are generally similar to *L.obscura* with rudimentary pseudocardinal teeth and greatly reduced or absent lateral teeth. However, *A.uwharriensis* is characterized by pronounced, somewhat curved pseudocardinal teeth in both valves and a well-defined interdental projection.

##### Phylogenetic placement.

*Ligodontaobscura* was inferred as the sister lineage to *Alasmidonta* in most phylogenetic analyses (Fig. 2; Suppl. material 1). However, the placement of *Ligodonta* was not well supported (<95% UFBoot) in most analysis and only moderately well supported on the COI tree (UFBoot 92%, see Suppl. material 1). Aside from an absence of morphological features that unite *Ligodonta* with other genera, the uncertain phylogenetic placement is also justification for erecting a new genus. Moreover, given the long branch that *L.obscura* is on, additional data is extremely unlikely to suggest that *Ligodonta* is a synonym of another genus. The long branch may be contributing to the uncertain placement of *Ligodonta*. We do not anticipate finding additional taxa (i.e. other undescribed species) that could be used to increase taxon sampling and shorten the branch in phylogenetic inference. Therefore, additional data, likely requiring slower evolving genes, may be necessary to determine where *Ligodonta* resides within the Anodontini phylogeny. This will also be critical for future studies examining biogeography, morphology, and life history evolution of the Anodontini.

##### Additional comparisons.

Several other mussel species native to the Uwharrie region are superficially similar to *L.obscura*. *Strophitusundulatus* Say, 1817 is a wide-ranging, though relatively uncommon species, found throughout Central and Eastern North America that possesses similarly reduced tooth morphology, but it differs from *L.obscura* by having a smooth peristrocum, single-looped beak structure, low posterior margin, and attaining a much larger maximum size. Young individuals of the common species *Uniomeruscarolinianus* Bosc, 1801 and *Elliptiocomplanata* are both characterized by a ventrally compressed shape and thin, papery periostracum and may resemble adult *L.obscura*, but are easily distinguished by a thickened shell, prominent sculptured pseudocardinal teeth, articulated lateral teeth, and pale white feet. Additionally, *U.carolinianus* has a single looped umbo structure and *Elliptio* species have a distinctive barred double looped umbo structure. *Toxolasmapullus* Conrad, 1838 is a small species with a patchy distribution and is sympatric with *L.obscura*, but is characterized by well-developed pseudocardinal and lateral teeth, double ridges on the posterior slope, a thickened and more ventrally inflated shell, and a rounded ventral margin.

*Ligodontaobscura* is known to co-occur with the following mussel species: *Alasmidontauwharriensis*, *Elliptiocomplanata*, *E.icterina* Conrad, 1834, *E.producta* Conrad, 1836, *Fusconaiamasoni* Conrad, 1834, *Sagittuniovaughanianus* Lea, 1838, *Strophitusundulatus*, *Toxolasmapullus*, *Uniomeruscarolinianus*, *Venustaconchaconstricta* Conrad, 1838, and *Villosadelumbis* Conrad, 1834.

## ﻿Discussion

Our results provide sufficient evidence to support the recognition of a new taxon endemic to the Uwharrie region of central North Carolina. *Ligodontaobscura* is easily distinguishable using morphological traits, genetic analysis, and biogeographic information. Historically, *L.obscura* has been misidentified as both *L.subviridis* and *A.varicosa*. The external shell characteristics of *L.obscura* superficially resemble other species, in particular *L.subviridis*, while internal shell characters are more closely aligned with some species of *Alasmidonta*. However, phylogenetic analyses make clear that *L.obscura* is not a member of either lineage.

Our survey results and museum records indicate *L.obscura* is a narrowly endemic species with a historical distribution in only two nearby Yadkin-Pee Dee River basin streams in central North Carolina. The Uwharrie region and nearby Sandhills support several endemic flora and fauna, including the recently described mussel, Uwharrie elktoe (*Alasmidontauwharriensis*). Every contemporary live specimen (*n* = 43) of *L.obscura* was collected from the Little River in Randolph County, North Carolina. The species was historically collected in nearby Barnes Creek (approx. 13 km linear distance; 120 river km), and may continue to persist in low densities, but was not recovered during our study. As currently understood, *L.obscura* has a restricted range of a single inhabited 7 km stream reach. *Ligodontaobscura* is therefore a likely candidate for conservation protections at the highest levels.

## Supplementary Material

XML Treatment for Ligodonta

XML Treatment for Ligodonta obscura

## ﻿Additional information

### Use of AI

No use of AI was reported.

### Author ORCIDs

Michael A. Perkins https://orcid.org/0000-0001-9068-8993

Nathan V. Whelan https://orcid.org/0000-0002-3542-2102

Heather K. Evans https://orcid.org/0000-0001-7090-3879

### Data availability

All of the data that support the findings of this study are available in the main text or Supplementary Information.

## Supplementary materials

Supplementary material 1 *Ligodontaobscura* supplemetary genetic datasets Data type zip Explanation note Datasets contain: Alignments in phylip formats (.phy); partition files that delineate starting blocks used for partitions (.txt). Can also be used to determine gene boundaries in concatenated datasets; best fit models and partitions inferred by ModelFinder (.nex). NOTE that the ITS-only analysis did not use partitioning, so there is only the alignment file for the ITS dataset.Tree-files contain: Tree inferred by IQ-TREE with each dataset. Trees are in Newick format. Tree files can be visualized by loading them into any phylogenetic tree viewer such as FigTree (https://github.com/rambaut/figtree/releases) or the web browser tool icytree (www.icytree.org). https://binary.pensoft.net/file/1390095This dataset is made available under the Open Database License (http://opendatacommons.org/licenses/odbl/1.0/). The Open Database License (ODbL) is a license agreement intended to allow users to freely share, modify, and use this Dataset while maintaining this same freedom for others, provided that the original source and author(s) are credited.Nathan V. Whelan
